# Small Bowel Endoscopy Diagnostic Yield and Reasons of Obscure GI Bleeding in Chinese Patients

**DOI:** 10.1155/2014/437693

**Published:** 2014-08-11

**Authors:** Ya-Fei He, Ning-Bo Hao, Wu-Chen Yang, Li Yang, Zhong-Li Liao, Chao-Qiang Fan, Jin Yu, Jian-Ying Bai, Shi-Ming Yang, Hong Guo

**Affiliations:** Department of Gastroenterology, Xinqiao Hospital, Third Military Medical University, Chongqing 400037, China

## Abstract

*Aim*. To investigate the diagnostic yield and etiologies of patients with obscure gastrointestinal bleeding (OGIB) using capsule endoscopy (CE) or double-balloon enteroscopy (DBE). *Method*. We studied the data of 532 consecutive patients with OGIB that were referred to Xinqiao Hospital in Chongqing from December 2005 to January 2012. A lesion that was believed to be the source of the bleeding (ulceration, mass lesion, vascular lesion, visible blood, inflammation, or others) was considered to be a positive finding. We analyzed the diagnostic yield of CE and SBE and the etiologies of OGIB. *Result*. CE and SBE have similar diagnostic yields, at 71.9% (196/231) and 71.8% (251/304), respectively. The most common etiology was erosions/ulceration (27.1%) followed by mass lesion (19.4%) and angiodysplastic/vascular lesions (13.9%). By stratified analysis, we found that erosions/ulceration (27.1%) was the most common etiology for the 21–40-year age group. Mass lesion was the most common etiology in the 41–60-year age group. However, in the >60 years age group, angiodysplastic/vascular lesions were significantly increased compared with the other groups, even though erosions/ulceration was most common. *Conclusion*. In this study, we found that CE and SBE have similar diagnostic yields and erosions/ulceration was the most common reason for OGIB, followed by mass lesion and angiodysplasias.

## 1. Introduction

Obscure gastrointestinal bleeding (OGIB) is defined as recurrent or persistent bleeding or iron deficiency anemia after a negative initial evaluation by gastric and colonic endoscopy [1]. It has been reported that OGIB is responsible for 5% of all gastrointestinal bleeding and that most of the lesions are in the small bowel [2].

In the past, the conventional diagnostic strategies for small intestine disease including small intestine radiography, abdominal computed tomography (CT), angiography, and red blood cell scanning have had a low diagnostic rate because of the length and unique anatomical structure of the small bowel [2–6]. Recently, with the development of capsule endoscopy (CE) and double-balloon enteroscopy (DBE), the study of the small bowel has been revolutionized. It has been demonstrated that CE is superior for detecting abnormal lesions noninvasively, with a higher rate of complete small bowel examination, and SBE is superior for endoscopic treatment [7, 8]. So CE and DBE are complementary methods for OGIB.

In previous studies, the main etiology for OGIB was considered to be angiodysplastic lesions [9, 10]. However, recent studies have suggested that this was true only in western populations and that ulceration was the most common etiology in Asian populations [9, 11, 12]. In this study, 532 patients with OGIB in our hospital from 2006 to 2012 were examined by CE or DBE and the etiologies were retrospectively analyzed.

## 2. Methods

### 2.1. Patients

OGIB was defined as overt bleeding (hematemesis, hematochezia, or melena) or occult bleeding (positive fecal occult blood test, iron deficiency anemia, or an acute drop in hemoglobin) in a patient with no pathologic causes that could be identified on conventional endoscopies. The exclusion criteria were as follows: serious physical condition, suspected perforation of the GI tract, bleeding tendency, and a lack of informed consent. Between December 2005 and January 2012, 545 consecutive patients who underwent CE and/or DBE for the indication of OGIB at the Department of Gastroenterology (Xinqiao Hospital, Third Military Medical University) were evaluated for inclusion and 532 patients that underwent CE or DBE were finally included. The studies had got the informed consent of all the patients included. Choosing CE or DBE for the examining was determined by the patients with the suggesting of doctors.

### 2.2. CE

CE studies (OMOM Jinshan Science and Technology (Group) Co., Ltd., Chongqing, China) were performed according to the standard protocol. Patients were asked to fast overnight after ingestion of 2 L polyethylene glycol-electrolyte solution before ingesting the capsule. Two hours after capsule ingestion, patients were allowed to drink and after 4 hours they were allowed to eat. Sensor array and recorder techniques were performed periodically to check the position of the capsule, and 8 h after ingestion the sensor array and recorder were disconnected. Data were then downloaded. All videos were reviewed by two experienced endoscopists who had each performed more than 100 capsule examinations.

### 2.3. Double-Balloon Enteroscopy

DBE was performed using a DBE system (Fujinon-Toshiba ES System, Saitama, Japan). This technique consisted of a video endoscope with an inner-diameter biopsy channel of 2.2 mm (EN-450P5) or 2.8 mm (EN-450T5), a flexible overtube, and a balloon controller. The DBE was performed through the mouth or colon according to the suspected site of the lesions. When the location was not clear, it was always performed through the mouth. The preoperative preparation, sedation, and analgesia were performed as described by Ohmiya et al. [13]. DBE was performed by two to three endoscopists at a time. Each had successfully performed the procedure at least forty times before the start of the study.

### 2.4. Statistical Analysis

Values are presented as medians, means ± SD, or percentages. For comparison of percentages, the *χ* ^2^ test and Fisher's exact test were used together with the calculation of the odds ratio and its 95% CI. The diagnostic yields and total CE and SBE rate were examined by *χ*-squared test. *P* values of less than 0.05 were considered to be statistically significant. All statistical analyses were performed using the Statistical Software Package version 11.0 (SPSS Inc., Chicago, IL).

## 3. Result

Overall, 545 patients were considered for OGIB and underwent CE or DBE. Among them, 235 patients underwent CE but 4 patients did not complete the procedure. Nine of the 310 patients undergoing SBE were excluded because they did not complete the procedure. Finally, 532 patients were included in the analysis. Two hundred thirty-one patients underwent CE and 301 patients underwent DBE. The basic characteristics of the patients are shown in Table 1.

As shown in Table 1, CE and DBE have similar diagnostic yields, which were 71.9% and 71.8%, respectively. Among these 532 patients, 382 patients (71.8%) had a positive result, 64 patients (12.1%) had a suspicious examination, and 86 patients (16.1%) had a negative examination. In both CE and DBE examinations, the lesion occurrence in the jejunum was similar to the ileum. However, CE examination had a higher detection rate when the lesions were diffuse (21.6% versus 16.3%). On the contrary, DBE had a higher detection rate in the duodenum (10.3% versus 3.5%).

Positive/suspicious lesions in patients with obscure gastrointestinal bleeding were as follows: mass lesion, bleeding, erosions/ulceration, angiodysplastic/vascular lesions, parasitic diseases, inflammation, polyps, and others (diverticulum and lymphangiectasis). As shown in Table 2, the most common etiology was erosions/ulceration (27.1%). Mass lesion (19.4%), angiodysplastic/vascular lesions (13.9%), and inflammation (11.0%) also occurred at high frequency. In addition, 5.6% of the patients showed bleeding in the endoscopy but the reason remained unknown.

By stratified analysis, it was found that in different age groups the etiologies were not the same. In the youngest age group (<20 years) the percentages of mass lesions, erosions/ulceration, inflammation, and polyps leading to bleeding were almost the same. In addition, 12% of the patients in this group were seen to be bleeding in the enteroscopy but the reason was not found, which is significantly higher than in the other groups. In the young age group (21–40 years), the most likely reason for bleeding was erosions/ulceration. Mass lesions and inflammation were also more common than other reasons. In the middle age group (41–60 years), the most significant reason was mass lesion, which was even a little higher than erosions/ulceration. In addition, angiodysplastic/vascular lesions and inflammation were also relatively common reasons for bleeding. In the old group (>60 years), erosions/ulceration (26.7%) was the greatest reason. But the occurrences of angiodysplastic/vascular lesions were significantly increased compared with the other groups. In summary, in this subgroup analysis, we found that, in the youngest group, the reason for bleeding was diverse, since the frequencies of the etiologies were similar. In the young age group, erosions/ulceration was the most common reason for bleeding. In the middle age group, mass lesion was the most common reason for bleeding. In the old age group, both erosions/ulceration and angiodysplastic/vascular lesions occurred more frequently.

In another stratified analysis, as shown in Table 3, we found that erosions/ulceration and mass lesion were the main reasons for bleeding in both males and females. However, in females, the occurrence of angiodysplastic/vascular lesions was much higher compared with males.

## 4. Discussion

CE and DBE have gained widespread clinical acceptance in the OGIB diagnostic process [7, 14, 15]. In the present study, we have reported on the diagnostic yield of these methods and the etiology in 532 patients with OGIB in the southwest of China. The main information obtained from this study was that the diagnostic yields for significant lesions by CE and DBE were similar (71.9% versus 71.8%). This is not consistent with a previous study, which has reported that the diagnostic yield of CE was significantly higher than a single DBE examination done via the oral or anal route (137/219 versus 110/219, OR: 1.67, 95% CI: 1.14–2.44, *P* < 0.01) [16]. The main reason for this difference may be because the subjects examined by DBE had more overt bleeding. It has been reported in a series of 260 patients with OGIB that the diagnostic yield was 87% in patients with ongoing overt OGIB and 46% in those with occult OGIB [17].

Previous studies have reported that angiodysplastic/vascular lesions were the most common cause of OGIB in western populations. Heine et al. reported that, in 168 patients with suspected small bowel bleeding, 123 (73%) had positive findings and the majority of cases involved angiodysplasia (52%) [10]. May et al. reported that, in 137 patients with suspected small bowel diseases, 109 (80%) had positive findings and the majority of cases involved angiodysplasia (37%) [9]. In the present study, 382 (71.8%) patients had positive findings. However, the most common etiology was small bowel erosions/ulceration (27.1%), followed by mass lesions (19.4%) and angiodysplastic/vascular lesions (13.9%). This is consistent with other Asian studies. Studies from Thailand, India, and Japan all showed that small bowel ulcers were the most common cause of OGIB (41%–53%), more common than angiodysplasia (23%-24%) [8, 12, 18, 19]. However, the rates are higher than ours, which may be because our study has a particular classification of the etiology. In addition, we also found that 7.6% of OGIB patients were induced by parasite disease. This was similar to a previous study which had reported that a high occurrence of parasite disease induced OGIB was found in China compared with others [20].

In the stratified analysis, we found that the causes of OGIB in the youngest group were diverse and the percentages of erosions/ulceration, mass lesions, inflammation, and polyps were similar. However, in the young group, the occurrence of erosions/ulceration was significantly increased (36.9% versus 18.2%, *P* < 0.05). In the middle group, mass lesions were the main cause. This is consistent with a previous study, which reported that, in patients between 40 and 60 years, tumors accounted for the largest proportion of OGIB [21]. In the old group, we found that the percentage of mass lesions was decreased, while the percentage of angiodysplastic/vascular lesions was increased just behind the amount of erosions/ulceration. This has also been demonstrated by Papadopoulos et al., who found that older patients had significantly less erosions and normal studies, but they had more angiodysplasias [11].

In addition, we also found that the percentages of mass lesion, erosions/ulceration, and inflammation had no significant difference between females and males. But the percentage of angiodysplasias in females was much higher than in males (19.0% versus 9.7%, *P* < 0.05). Furthermore, we also found that, in both female and male groups, more than half of the patients were 60 or older; this indicated that we should pay more attention for the angiodysplasias in old patients. Overall, in the present study, we found that the etiologies were not similar between females and males or young and old patients.

The large sample number was one strength of the present study, but it was not without limitations. Firstly, the endoscopists reported mucosal erosions of the small bowel, but further definitive categorization of such lesions was not clear. Secondly, the follow-up results for the patients with positive findings were unknown.

In conclusion, we found that erosions/ulceration was the most common reason for OGIB, followed by mass lesion and angiodysplasias. The etiology for OGIB in different age groups was not similar. In the 21–40-year age group, erosions/ulceration was thought to be the main reason; in the 41–60-year age group, the percentage of mass lesions increased. In >60 years age group, angiodysplasias were not the primary reason but had significantly increased over the others. In females, angiodysplasias also had a high frequency of occurrence compared with males.

## Figures and Tables

**Table 1:** Clinical characteristics, indication, and finding results for CE and SBE.

Characteristics	Total	CE	SBE
	532	231	301
Mean age (y)	50.0 ± 18.3	54.5 ± 17.7	46.6 ± 18.1
Sex (female)	232 (43.4)	115 (49.8)	117 (38.9)
Indication			
Occult bleeding	17	12	5
Overt bleeding	515	219	296
Results			
Positive	382 (71.8)	166 (71.9)	216 (71.8)
Negative	86 (16.1)	35 (15.1)	51 (17.0)
Suspicious	64 (12.1)	30 (13.0)	34 (11.2)
Location of findings			
Duodenum	39 (7.3)	8 (3.5)	31 (10.3)∗
Jejunum	144 (27.1)	64 (27.7)	80 (26.6)
Ileum	134 (25.2)	58 (25.1)	76 (25.2)
Diffuse	99 (18.6)	50 (21.6)∗	49 (16.3)
Others∗	30 (5.6)	16 (6.9)	14 (4.7)

Others include esophagus, stomach, and colon bowel.

∗Significant between CE and DBE in the same location of findings.

**Table 2:** Positive/suspicious lesions in patients with obscure gastrointestinal bleeding (*n* = 446).

	Total (%)	<20	21–40	41–60	>60
Erosions/ulcerations	121 (27.1)	6 (18.2)	41 (36.9)	34 (22.3)	40 (26.7)
Mass lesion	86 (19.4)	5 (15.2)	19 (17.1)	38 (25.0)	24 (16.0)
Angiodysplastic/vascular lesions	62 (13.9)	2 (6.1)	10 (9.0)	17 (11.2)	33 (22.0)^1^
Inflammation	49 (11.0)	5 (15.2)	13 (11.7)	17 (11.2)	14 (9.3)
Polyp	39 (8.7)	5 (15.2)	8 (7.2)	16 (10.2)	10 (6.7)
Parasitic diseases	34 (7.6)	2 (6.1)	7 (6.3)	11 (7.2)	14 (9.3)
Blood on CE or SBE	25 (5.6)	4 (12.0)	6 (5.4)	7 (4.6)	8 (5.3)
Others	30 (6.7)	4 (12.0)	7 (6.3)	12 (7.9)	7 (4.7)

Total	446	33	111	152	150

Others included diverticulum and lymphangiectasis. ^1^ *P* < 0.05, compared with the patients with angiodysplastic/vascular lesions in 41–60-year age group.

**Table 3:** Positive/suspicious lesions in patients with obscure gastrointestinal bleeding (*n* = 446).

	Total (%)	Female (*n* = 196)	Male (*n* = 250)
Erosions/ulcerations	121 (27.1)	48 (24.0)	73 (29.7)
Mass lesion	86 (19.4)	39 (19.5)	47 (19.1)
Angiodysplastic/vascular lesions	62 (13.9)	38 (19.0)∗	24 (9.7)
Inflammation	49 (11.0)	21 (10.5)	28 (11.4)
Polyp	39 (8.7)	14 (7.0)	25 (10.2)
Parasitic diseases	34 (7.6)	16 (8.0)	18 (7.3)
Blood on CE or SBE	25 (5.6)	11 (5.5)	14 (5.7)
Others	30 (6.7)	13 (6.5)	17 (6.9)

Others included diverticulum and lymphangiectasis. *P < 0.05, compared with the percentage of angiodysplastic/vascular lesions in males.
